# Promises and pitfalls in implementation science from the perspective of US-based researchers: learning from a pre-mortem

**DOI:** 10.1186/s13012-022-01226-3

**Published:** 2022-08-13

**Authors:** Rinad S. Beidas, Shannon Dorsey, Cara C. Lewis, Aaron R. Lyon, Byron J. Powell, Jonathan Purtle, Lisa Saldana, Rachel C. Shelton, Shannon Wiltsey Stirman, Meghan B. Lane-Fall

**Affiliations:** 1 Philadelphia, USA; 2 Seattle, USA; 3 St. Louis, USA; 4 New York, USA; 5 Eugene, USA; 6 Menlo Park, USA

**Keywords:** Implementation science, Pre-mortem exercise, Threats, Opportunities

## Abstract

**Background:**

Implementation science is at a sufficiently advanced stage that it is appropriate for the field to reflect on progress thus far in achieving its vision, with a goal of charting a path forward. In this debate, we offer such reflections and report on potential threats that might stymie progress, as well as opportunities to enhance the success and impact of the field, from the perspective of a group of US-based researchers.

**Main body:**

Ten mid-career extramurally funded US-based researchers completed a “pre-mortem” or a group brainstorming exercise that leverages prospective hindsight to imagine that an event has already occurred and to generate an explanation for it — to reduce the likelihood of a poor outcome. We came to consensus on six key themes related to threats and opportunities for the field: (1) insufficient impact, (2) too much emphasis on being a “legitimate science,” (3) re-creation of the evidence-to-practice gap, (4) difficulty balancing accessibility and field coherence, (5) inability to align timelines and priorities with partners, and (6) overly complex implementation strategies and approaches.

**Conclusion:**

We submit this debate piece to generate further discussion with other implementation partners as our field continues to develop and evolve. We hope the key opportunities identified will enhance the future of implementation research in the USA and spark discussion across international groups. We will continue to learn with humility about how best to implement with the goal of achieving equitable population health impact at scale.

**Supplementary Information:**

The online version contains supplementary material available at 10.1186/s13012-022-01226-3.

Contributions to the literature

- We completed a pre-mortem exercise to identify potential threats that might stymie the field of implementation science.
- We identified six themes that offer opportunities for future success for the field.
- Opportunities include pragmatism, balancing scientific rigor with adapting to and prioritizing what clinical and community partners need, and being ready to act rapidly when the context demands.

## Background

Research on dissemination and implementation has a long, rich history [1]. We are grateful to be a part of that history as some of the first US researchers to build implementation science careers as the field was formalizing [1]. Our backgrounds are in psychology, public health, social work, education, and medicine with foundations in intervention science, clinical science, community psychology, and health services research. Most of us have clinical training and experience. We came to implementation science frustrated because patients and community members did not routinely receive evidence-based practices (EBPs) and because policies were not aligned with high-quality research evidence. We became aware that resources spent developing EBPs were not translating into their routine delivery outside of research contexts, and recognized that racially, ethnically, and socioeconomically diverse communities, populations, and settings that would benefit most from EBPs were not equitably reached. Implementation science attracted us as a way towards equitably changing healthcare quality, systems, and outcomes [2]—that is, achieving population health impact and social justice at scale [3].

Implementation science has reached an appropriate time developmentally to reflect on its progress. In 2006, the flagship journal *Implementation Science* was launched. The first National Institutes of Health (NIH) implementation science conference was held in 2007. The past 15 years have seen thousands of articles; funding mechanisms, including from the NIH, the UK Medical Research Council, and the Canadian Institutes of Health Research; international meetings; additional journals; and a growing global cadre of implementation scientists.

In recent years, there have been several self-critical assessments of the field, made by leading implementation scientists [4–9]. These self-critical assessments are welcomed because they challenge commonly held assumptions and present opportunities to move the field forward. In reviewing the literature, we would like to see even more of these discussions given recent concerns that the field may be stagnating [4, 6]. First, the majority of these self-critical assessments have been led by teams outside of the US, including Europe [4], Australia [5], and Canada [6]. In this commentary, we offer a US-based perspective on the opportunities for the field to continue to have forward momentum. Second, many of these assessments are based upon retrospective reflections from leading implementation scientists. To come to consensus on the themes that we shared in this commentary, we used the innovative pre-mortem technique. A pre-mortem uses prospective hindsight—a group imagines a failure and generates an explanation for it—to reduce the likelihood of the failure [10] which allows for leveraging prospective hindsight to prospectively generate potential threats to the field rather than the retrospective approach of a post-mortem (see Additional file 1 for more details on the approach). Third, while two of the self-critical assessments from Europe [4] and Australia [5] offer similar perspectives, particularly around partner engagement and the importance of embedded research infrastructures and capacity building, our commentary builds upon these assessments and presents a set of threats and opportunities not fully articulated in the previous pieces, particularly not from the perspective of what the potential outcome might be if the field cannot address existing threats. Other self-critical assessments are focused on specific issues within the field such as new directions in audit and feedback [6], how implementation science might be relevant in COVID-19 [7], the cultural politics of the field [9], and personal reflections of one senior author [8]. Finally, our commentary offers the perspective that implementation science as a discipline is not immune from the critiques of other sciences which is not explicitly stated in previous self-critical assessments. We hope this commentary will inspire dialogue, new solutions and methods, and innovative partnerships across international teams and highlight the only partially tapped utility of implementation science for improving population health equitably.

## Main body

The six themes we discuss below which were identified through the pre-mortem (Table 1) are also challenges in the fields in which we initially trained. These themes threaten forward movement if we are not thoughtful about the field’s evolution and growth. Framing these themes using prospective hindsight highlights their complexities and points toward potential opportunities.

**Table 1:** Threats that might stymie progress in the field of implementation science: Themes, causes, and solutions

Pre-mortem threat theme	Cause of threat	Solutions to address threat
We did not impact population health or health equity	• Structural factors have only recently become a focus in the field of IS, despite being critical to population health and health equity impacts • It takes a long time and substantial resources to change determinants of implementation at multiple levels, and these changes are critical to population health and health equity impacts • Misalignment between metrics used to judge IS researcher success (e.g., publications, grants) and metrics of population impact	• Document impact at organizational, community, and policy levels in IS publications • Efficiently include health outcomes in IS studies by leveraging routinely collected heath outcome data and other pragmatic measures • Use multidisciplinary team science approaches in IS to address structural and social determinants of implementation • Prioritize focus on implementation strategies and EBPs that have the most potential to promote health equity • Routinely measure the impacts of implementation strategies on health equity outcomes • Generate more evidence about associations between implementation outcomes and health outcomes, the impacts of implementation efforts on health equity outcomes, and economic return on investment of implementation strategies • Translate findings from IS research into useable tools for practitioners and clearly communicate the value of IS to the public and policymakers
We over anchored on becoming a “legitimate” science	• Lack of critical thinking about which IS frameworks to use and IS outcomes to focus on for a specific context • Holding too firmly to the belief that there needs to be an EBP—as opposed to a program that meets a community’s need but has not yet demonstrated effectiveness—in order to conduct IS • Tendency to borrow from fields and disciplines outside of IS to maximize innovation without fully leveraging the body of knowledge in those areas	• Embrace critiques of the field of IS and reflect on them to advance thinking and the field • Focus on adapting existing frameworks, theories, and outcomes within IS to place greater emphasis on health equity • Bring in experts from adjacent disciplines and strive for transdisciplinary approaches to meaningfully innovate
We recreated the research-to-practice gap	• Prioritizing researcher over practitioner perspectives in IS and prioritizing the production of generalizable knowledge over knowledge that has practical applications in a “real word” context • Many IS frameworks and methods lack utility for practical application beyond a research study	• Refine IS frameworks to place greater emphasis on pragmatism and application • Develop modular implementation strategies that can be easily tailored and have broad applicability across practice settings • Better integrate the perspectives of researchers and practitioners and promote opportunities for meaningful collaboration
We could not balance making implementation science available to everyone while retaining the coherence of the field	• Implementation scientists act as gatekeepers to the field because they seek to establish IS as a coherent field	• Consider the IS training needs of three different groups: (1) those who should have general awareness of IS but are unlikely to incorporate it into their work, (2) those who will use IS as part of their broader toolkit, and (3) those who will narrowly focus on IS and advancing the field • Create divisions and departments of IS within institutions, comprised of individuals with extensive IS expertise who apply it to help address implementation challenges/research questions across the institution
We could not align our timelines, incentives, or priorities with our partners	• By the time a IS research study is complete, the practice context surrounding implementation has changed and there is a misalignment between the knowledge produced and the context in which it was intended to be applied • The incentive structures for researchers—imposed by universities, funders, and health systems—are often misaligned with those of the community partners needed to conduct impactful IS	• Embrace an embedded IS researcher model that prioritizes partner needs and priorities over researcher interests and co-creates evidence together • Co-design funding mechanisms that are aligned with community timelines and needs • Use rapid IS methods
Our implementation strategies and processes were too complex and not well matched to partner needs	• Taxonomies of implementation strategies and behavior-change methods and techniques are not exhaustive and may not be readily understandable by community partners • Pressure to prioritize innovation can lead to rebranding and reinventing the wheel rather than conceptual and empirical investment • The language of “implementation mechanisms” may make our science feel less relevant to community-based collaborators • Methods to design, select, and tailor implementation strategies can be resource intensive, leading to partner burden	• Embrace approaches that prompt implementers to consider what multilevel changes are required to implement, scale, and sustain interventions; what might help or hinder those changes; and how changes can be feasibly measured • Determinants could be more feasibly identified through systematic reviews, rapid approaches to contextual inquiry, or existing data, and approaches to rapidly prioritize determinants could be identified and evaluated • Prioritize pragmatic approaches to implementation strategy design, selection, and tailoring • Create and improve tools to help implementers identify, develop, and apply implementation strategies • Consider the mechanisms through which strategies operate to ensure that they are designed as efficiently as possible and to inform adjustments for poorly performing strategies • Leverage user-centered designs and approaches such as the Multiphase Optimization Strategy (MOST), Learn as You Go (LAGO), and Sequential Multiple Assignment Randomized Implementation Trial (SMART) designs that prioritize adaptation and efficiency • Improve reporting of implementation strategies to avoid pseudoinnovation and move toward the identification of “common elements” of effective implementation strategies • Embrace approaches to efficiently build the evidence for implementation strategies, such as the Audit and Feedback Metalab [6, 11]

*IS* implementation science, *EBP* evidence-based practice

### Theme 1: We did not impact population health or health equity

#### Threats

Impact is foundational to implementation science. We considered impact from the equity perspective of deploying discoveries that are relevant, appropriate, and feasible across diverse populations and settings for widespread health and societal benefits, while acknowledging the complexity of defining impact [12]. The literature has only a few examples of the field having broad impact (e.g., implementation of patient safety checklists) [3]. This scarcity of success may be due to many implementation studies having null results, implementation efforts taking many years to influence public health, or a misalignment between reporting impact broadly and metrics such as papers and grants used to evaluate researchers and the quality of their research [13]. Regardless, as the field coalesces and grows, funding, uptake, and scaling of implementation approaches require that they demonstrate societal and population health impact and economic value. Below, we outline tensions we can address to demonstrate impact as the field continues to develop and demonstrate its utility [14, 15].

Our mission to improve EBP implementation is more complex than instituting a discrete strategy [16]. The field’s relatively focused endeavor to improve the widespread, routine adoption, implementation, and sustainment of EBPs has therefore evolved to be more all-encompassing. This is partly attributed to findings that organizational factors such as culture predict much of the ability of health service organizations to provide high-quality care and implement EBPs [17, 18] and that policy significantly shapes health and inequities, partially through financing and incentives for changing the healthcare status quo [19]. Additionally, as part of context, upstream societal and structural factors such as structural racism and social determinants of health are recognized as critical for shaping health inequities and inequitable implementation [20]. Only recently, however, has the field more explicitly included and measured these determinants and their impact on implementation and health outcomes [20–23]. Given the important role of multilevel context in implementation, understanding the real-world complexity and interconnected nature of these determinants is critical. Yet inclusion of these complexities in our models and solutions takes more time and resources than was originally thought for a field whose mission is to hasten the deployment of science to practice.

#### Opportunities

As implementation researchers, our publications ought to detail impact, both with empirical evidence about health outcomes (including whether outcomes were equitably improved in all groups) in our studies and impact at organizational or policy levels resulting from research and partnerships (e.g., if results led to state funding for EBP delivery or partners report that implementation challenges were addressed). Measuring health outcomes is often challenging when study resources are allocated to rigorous evaluation of implementation strategies and outcomes, but may offer the greatest opportunity to demonstrate impact. Increasingly, we need to leverage routinely collected heath outcome or administrative data and other pragmatic measures [24].

Another potential solution to increase impact is better defining an implementation strategy’s scope. Some focus on proximal implementation and clinical outcomes and should acknowledge an inability to meaningfully impact system-level outcomes; others are designed for system-level effects and should state limitations for individual impact. This suggestion stems from our experience studying individual clinician behavior to state and national policies, and realization that balancing breadth and depth is important for the future of implementation. It also underscores the importance of being explicit about how and why an implementation strategy is intended to work (i.e., specifying hypothesized mechanisms) [16, 25, 26].

Because of the need to consider context, multilevel system variation, and other complexities while accelerating the implementation of EBPs in communities, team science is essential [27] for equitable impact. Examples include applying implementation science to examine and address social and structural determinants (e.g., structural racism) as part of contextual assessments to advance understanding of barriers to implementation or informing selection or refinement/adaptation of EBPs and/or implementation strategies [20, 28]. This work, in collaboration with community members and leaders, intervention developers, prevention scientists, policymakers, and other scientific and practitioner partners, can provide a foundation and strategies for shared responses to inequities or uneven EBP implementation informed by implementation and policy development focused on and prioritizing health equity [29, 30]. Implementation scientists can also prioritize EBPs and strategies with potential to promote health equity to highlight the value and impact of the field and avoid inadvertently reinforcing inequities. We can measure and track equitable delivery of EBPs and implementation strategies across populations and settings and the extent that approaches alter health inequities [20, 21].

Areas in which we ought to generate more evidence to demonstrate impact include (a) investigating the relationship between our implementation outcomes and health outcomes [31] and prioritizing both sets of variables such as suggested by hybrid designs [16]; (b) demonstrating improvement in population health, including in promoting health equity and reducing health inequities [22]; and (c) demonstrating the economic impact of EBP implementation and of poor/ineffective implementation [14] (i.e., return on investment and value). Demonstrating the economic costs of effective strategies is critical [14, 16, 32, 33]. Without compelling evidence that implementation science-informed approaches yield a favorable return, policymakers and administrators may be reluctant to invest time and resources in complex approaches. Identifying the best approach to economic analysis and ensuring collection of this data during implementation efforts is critical to building a business case for funding implementation.

Finally, and perhaps most importantly, translating our scientific knowledge into usable knowledge for the public is a way forward for impact. This can be accomplished through multiple avenues. The recently published National Cancer Institute practitioner guide to implementation science [34] is one example of a product that can translate research to practice. We recommend that implementation scientists also clearly communicate the value of the field to the public and policymakers. The COVID-19 pandemic underscores the value of an implementation science-informed approach: An influential simulation paper prior to emergency-use approval for COVID-19 vaccines suggested that implementation rather than vaccine effectiveness would be the major challenge to global population vaccination [35], precisely predicting the vaccine rollout challenges. If more people in the public and in healthcare knew what implementation science offers, we could have more impact and added value to public needs. As implementation scientists, our responsibility is to shape the narrative about the value of our field [36]. This includes communicating our work in understandable ways and answering the key questions that policymakers, the public, and our broader communities have for us in lay venues including op-eds [37, 38].

### Theme 2: We over anchored on becoming a “legitimate” science

#### Threats

The past 15 years have seen a flurry of activity around codifying and legitimizing the science of implementation. This pattern is consistent with the emergence of a new field with no common body of facts and scientists converging on conceptual frameworks, terminology, methods, and designs to answer research questions [39]. A shared lexicon and tools are laudable goals and can legitimize implementation science, but potentially undermine the future of the field if not approached thoughtfully.

First, we observe a tendency in the field to reify commonly used frameworks, approaches, and ways of thinking. Using similar terminology has clear communication advantages, but we see a disadvantage to all studies applying the same conceptual frameworks, designs, and methods without critical thinking, which can contribute to stagnancy and limit innovation. For example, while Proctor and colleagues’ influential 2011 paper substantially advanced the field by defining implementation outcomes [40], scholars rarely posit outcomes beyond this initial set. A few of the outcomes are over-represented (e.g., fidelity) compared to others.

A second example is the idea that implementation science-related inquiries require an EBP rather than simply an existing innovation or program that meets a community’s need [41]. The COVID-19 pandemic demonstrated how quickly implementation science might become obsolete if we only get involved when there is an EBP [42, 43]. Furthermore, approaches that over-prioritize scientific evidence over community-defined evidence can disempower community partners [41, 44]. This might manifest as EBPs that do not reflect or involve populations that experience historical or ongoing mistreatment, discrimination, or injustices from public health and/or medical institutions, presenting foundational challenges in our ability to equitably reach, implement, and sustain EBPs [21].

A third challenge is related to our borrowing from disciplines such as organizational theory, behavioral science, and systems science. One danger, since funders and reviewers prioritize novelty [45], is borrowing from other fields to maximize innovation but doing so in a superficial manner that does not reap the benefits of deep interdisciplinary or transdisciplinary work.

#### Opportunities

Healthy critiques, reflection, and dismantling of current thinking are needed for scientific field development. We have opportunities to innovate in our methodologies and theories before settling on what is “widely accepted” [46, 47]. Although we have 150 published implementation frameworks [48] and must carefully consider the value of adding more, frameworks are still opportunities to shift paradigms and advance theory. Deeper application and evolution of methods from other adjacent fields applied to implementation are opportunities to harness well-vetted theory, advance our science, and increase rigor and impact, particularly in promoting health equity. For example, we have seen recent innovations in adapting existing theories, models, and frameworks to focus more on equity (e.g., see [23, 49–51]). We note opportunities to learn from and integrate theories and frameworks from fields with a long history of health equity scholarship, including anthropology, sociology, and public health [52]. Simultaneously, we cannot overpromise the benefits of implementation science: We will quickly become disillusioned if we are not circumspect about the potential benefit — or lack thereof — of the products of our implementation work.

### Theme 3: We recreated the research-to-practice gap

#### Threats

Although implementation science was created to reduce the research-to-practice gap, recent critiques suggest we may be recreating it [53]. This could undermine the forward movement of the field [5], including work to reach populations experiencing health inequities [22]. More bidirectional partnership between implementation research and practice is needed [54].

Because implementation science requires multilevel and partnered approaches (theme 1), it is complex by nature. Input from multiple sources that often prioritizes researcher perspectives may lead implementation strategies to be developed without “designing for implementation.” In other words, many strategies are designed for maximal theoretical effect, with the unintended consequence of limiting fit, feasibility and/or affordability [55]. Additionally, because implementation science is relatively new, investigators may feel pressure to develop their own approach to push the field forward, especially given the premium that funders and reviewers place on innovation. The resulting innovation may be less responsive to partners and the result too complex or incompatible with many practice settings. There may be limited access to the implementation strategy due to limited capacity to train others in complex, “proprietary” strategies. As we have been advocating for intervention developers to design for implementation for years [56], we might consider heeding our own advice.

Second, the state of implementation frameworks is challenging because of both their number and their utility for pragmatic application. The multitude of implementation frameworks [48] creates considerable difficulty for researchers and community partners in selecting a framework to guide their work and pragmatically apply the findings.

Third, a key tension that we hear from partners is that implementation science should balance adaptation for context with generalizable knowledge. While context is key [57, 58], tailoring solutions for particular sites or efforts may not always be possible with limited resources. We ought to balance pragmatism, the creation of generalizable knowledge, and finite resources.

#### Opportunities

To avoid recreating the research-to-practice gap, we should balance advancing implementation science theory and general knowledge with serving research and community partners, all with finite resources. Solutions may include refining commonly used frameworks to enhance pragmatism and facilitate application. An example is the sixth domain added to the Consolidated Framework for Research (CFIR) focused on patient needs [59] and adaptation of CFIR for the context of low- and middle-income countries [59].

Developing modular (i.e., menu of common implementation strategies) implementation approaches is an opportunity for innovation and creating broadly useful strategies for tailoring to setting. These solutions are opportunities for both implementation researchers and practitioners, who apply research to transform practice. We can be partners in advancing knowledge quickly and ensuring rigor, relevance, and translatability. As humans, we are prone toward dichotomous thinking, but implementation science will be stronger if we prevent the emergence of separate “research” and “practice” ideologies. Another opportunity is a lesson from intervention development: avoid assuming “if you build it, they will come” [60].

To avoid a research-to-practice gap in implementation, we should assemble the voices of all key partners including community members, implementation researchers, and practitioners. The most effective way forward is true partnership to advance knowledge quickly and ensure rigor and relevance, rather than the emergence of “research” and “practice” camps separated by ideological lines. One solution comes from the Society for Implementation Research Collaboration (SIRC), which proposed an integrated training experience for implementation researchers, practitioners/intermediaries, practice leaders, and policy leaders to reduce the implementation research-practice gap [60]. Building on principles of pragmatic research, team science (theme 1), and interprofessional education, the approach could be a model for integrated professional development.

### Theme 4: We could not balance making implementation science available to everyone while retaining the coherence of the field

#### Threats

A major challenge of the field relates to capacity building [61], with the goal of making implementation science more broadly available to implementation research and practice. Pressures to create traditional niches of expertise have resulted in a sometimes insular field that often requires individuals to be perceived as “card-carrying” implementation scientists to obtain funds for large-scale implementation research. If we want to meet demand, have broader impact, and formalize as a scientific field, we need more implementation scientists [62]. However, having everyone “do implementation science” has the potential to dilute the field and lessen perceived innovation and coherent science. The epistemology of science has many theories on the tension of how fields grow and thrive [63]. If we, as implementation scientists, act as gatekeepers to retain field coherence, we lose opportunities to partner with adjacent fields such as improvement [64] and intervention sciences and grow synergistically, rather than in parallel and siloed. We give up the chance to embed in learning health systems [65] and other organizations available internationally in the US [66], UK [67], and Australia — and repeatedly proposed in low- and middle-income countries [68, 69] — for synergy between implementation science-informed approaches and quality improvement, clinical informatics, and innovation [70].

#### Opportunities

There is a growing understanding that more implementation science capacity is needed, while retaining field coherence [71, 72]. One way that we have come to think about this includes considering the needs of three groups. First, basic scientists and early-stage translational researchers should be aware of implementation science but will likely not incorporate its approaches into their work without partnering with an implementation scientist. This group benefits from awareness of implementation science methods, which can be built into graduate and/or postdoctoral training. The second group of individuals might include implementation science in their toolkit (e.g., health services researchers, intervention developers, clinical trialists) and use established methods (e.g., hybrid designs, co-design) in their projects. This group requires foundational training. The third group are dedicated implementation scientist methodologists and advance the field with their work. These individuals require advanced specialized training. They may be most interested in a particular disease (e.g., cancer) and setting (e.g., acute care or schools) or be disease and setting agnostic, instead answering the most impactful implementation science questions. The intentional development of these different groups will promote the full range of implementation science, from basic science focused on theory and method development to applied, real-world approaches [73].

We envision a future in which all institutions, including large health systems, have a division or department of implementation scientists from the third group. We envision departments having individuals with expertise in implementation science germane to the department’s area, akin to the biostatistician model [74, 75]. Exploration of additional models for supporting institutional implementation science capacity, including leveraging Clinical and Transitional Science Award programs [73, 76, 77], is needed. This kind of growth will both democratize implementation science and promote paradigm-shaping work needed to advance the field.

### Theme 5: We could not align our timelines, incentives, or priorities with our partners

#### Threats

Challenges in alignment with partners have been described in related fields [73, 78]. Meaningful partnership with care delivery and community settings is the backbone of implementation science [79–81]. Thus, to do implementation research well, we should invest in and maintain relationships with community partners and the systems that employ or serve them. We define community broadly to include administrators, staff, and clinicians from local and state governments, payers, payors, community-based organizations, and health systems, as well as community members, community leaders, community-based organizations, and patients and caregivers who are reached through them [82]. A major threat to the long-term viability of implementation science concerns alignment on timeline, priorities, and incentives between our partners and the scientific enterprise of implementation research.

First, the priorities and timeline for implementation research are often misaligned with health systems and community settings. Science can be slow [83], and once health system or community leadership sets priorities around healthcare delivery, they expect change quickly. Similarly, needs and priorities might not align with those proposed in implementation research, particularly if partners are not meaningfully integrated into the research process from the outset [82], or if inequitable power and resource dynamics exist. In addition, by the time research is completed, contexts may have shifted, new interventions may have been developed, and the lag in delivery of the most advanced healthcare solutions persists, especially for under-resourced settings.

Second, academic incentives and the transformation of health and healthcare have a fundamental tension. As is typical in academia, implementation scientists are incentivized to publish and apply for grants rather than to transform practice — widening the research-to-practice gap (theme 3). This is a longstanding issue for healthcare researchers and other academics with public or population health impact as their explicit goal [12, 84]. These alignment challenges are influenced and compounded by current funding mechanisms. This makes launching projects responsive to emergent needs challenging, creating disappointment or disillusionment in partners who are unfamiliar with grant timelines and processes.

#### Opportunities

We ought to move towards pragmatic implementation science which prioritizes the needs of partners [3, 85]. One model that mitigates this issue is embedded research [3], in which researchers work within settings such as health systems, funded by them to answer questions that match their priorities [86]. Instead of one-off studies, evaluation and research are built into implementation efforts for sequential, timely, rapid learning. This model allows both system change and creation of generalizable knowledge to transform other organizations and systems. An example is the creation of implementation laboratories such as the Audit and Feedback Metalab [6, 11]. This model might become the norm, not the exception. However, some care settings are chronically underfunded and under-resourced, including publicly funded mental health and smaller health service organizations, public health, and justice settings, likely limiting embedded research with them.

Another opportunity is funders and partners codesigning funding mechanisms that are responsive to the community timeline and needs and sufficient for rigorous evaluation and tangible impact. Funders have recently deployed more flexible mechanisms (e.g., National Cancer Institute Implementation Science Centers in Cancer Control [87], NIH COVID RADx initiative) by including mechanisms with more resources and support for community partners, but most traditional mechanisms typically do not align with real-world needs [88].

The power of alignment in implementation science is illustrated by the COVID-19 crisis with coalescing of political, community, public health, and health system priorities around COVID-19 prevention and care. Some implementation scientists pivoted to use rapid implementation science methods to meet their settings’ needs, exemplifying how to offer our skillset to partners in a time of need. For example, Penn Medicine used implementation mapping [89] to assemble key partners to develop five specific strategies for rapid implementation during the pandemic to improve prone positioning of patients to ameliorate COVID-19 symptoms [90]. One potential way to harness alignment is prioritizing rapid implementation science [91–93], which balances speed, efficiency, and rigor in implementation by adapting both methods and trial design to meet objectives [94, 95]. Rapid implementation methods will continue to gain traction if researchers and partners continue to prioritize efficiency in methods and designs while maintaining rigor.

### Theme 6: Our implementation strategies and processes were too complex and not well matched to partners’ needs

#### Threats

Implementation strategies have progressed tremendously, including ways to classify implementation strategies conceptually [96–98], generation of an increasingly robust evidence base from rigorous trials, and establishment of reporting guidelines to improve rigor and reproducibility [99–103]. Despite these advances, complexity and misalignment with needs threaten the application of implementation strategies.

First, despite conceptual advances, our taxonomies of implementation strategies and behavior-change methods and techniques are by no means exhaustive. Fields such as behavioral economics and systems engineering offer insights on how to shape clinician decision-making under conditions of uncertainty or develop approaches that match local needs, but these approaches are underemphasized in existing taxonomies such as the Expert Recommendations for Implementing Change (ERIC) compilation [17]. Moreover, many of our strategies are not readily understandable by community partners, as they have arisen out of predominately clinical contexts, and require translation for applicability in community settings.

Second, the pressure to innovate (themes 2 and 3) can lead to rebranding, testing, and promoting implementation strategies that reinvent the wheel or represent incremental improvements or tweaks from previous studies. Rebranding and tweaking eliminates advantages of shared language [104] (theme 2) and stymies conceptual and empirical development of the field.

Third, as the field focuses on understanding implementation strategy mechanisms [105], which helps us understand how strategies work and build causal theories, we risk becoming overly reductionist. Simply the language of “implementation mechanisms” may make our science feel less relevant to community-based collaborators. Our designs and methods also may stymie progress, for example emphasizing traditional designs such as randomized controlled trials rather than designs (e.g., adaptive, rapid, systems science-based [106]) suited to developing and determining if strategies have signals of effectiveness [107] or that capture dynamic, social processes within context.

Finally, our processes to design and tailor implementation strategies are imperfect and often a mismatch for the challenges of the partners and setting [4, 108]. While the basic steps of designing and tailoring implementation strategies systematically are documented [109, 110], the process of selecting implementation strategies often requires intensive contextual inquiry and the strategies that effectively address the identified implementation determinants are unclear. Not surprisingly, partners express frustration with the lengthy process, suggesting the need for methods that balance rigor and pragmatism.

#### Opportunities

Numerous ways may lead to development of implementation strategies that are better matched to determinants, more understandable to our partners and more pragmatic, and more efficiently build the science of implementation. First, we can embrace systematic approaches that prompt implementers to consider what multilevel changes are required to implement, scale, and sustain interventions; what might help or hinder those changes; and how changes can be feasibly measured [103, 109]. Approaches ideally incorporate existing partner input, evidence on strategy effectiveness, and formal or informal theory that hypothesizes mechanisms of strategy operation. Considering mechanisms can ensure that strategies are as efficient as possible and allow adjustment of poorly performing strategies in subsequent efforts [25, 26, 105, 111]. Systematic methods [110, 112] include intervention (or implementation) mapping, increasingly applied to systematically design and/or tailor implementation strategies [89, 113]. Ample opportunities remain to improve these and other methods to be more pragmatic and useful to implementers.

Implementation and sustainment determinants could be more feasibly identified through systematic reviews, rapid approaches to contextual inquiry [94, 114, 115], or existing data such as information included in the electronic health record. Determining how to rapidly prioritize determinants is also important. For example, one approach being tested involves evaluating the ubiquity, chronicity, and criticality of implementation determinants to prioritize which should be explicitly addressed [116].

Improving tools to help implementers identify appropriate implementation strategies is critical. This could involve refining taxonomies of implementation strategies such as the ERIC compilation to make the content and language more usable to partners in specific contexts (e.g., school mental health, community-based organizations) [100, 117, 118]; incorporating strategies from fields such as behavioral economics [17]; noting strategy relevance to specific phases (e.g., exploration, sustainment) and translational tasks (e.g., dissemination, scale-up) [119]; and articulating strategy mechanisms [105]. Tools that match strategies and behavior-change techniques to implementation determinants could help organizations and systems tailor strategies to their needs and be improved over time to incorporate conceptual and empirical advancements [117, 120].

Additional approaches to improve fit between strategies and partner needs and contexts include using and refining strategies that are inherently adaptive, such as facilitation [121, 122]. We could leverage user-centered design and approaches such as the Multiphase Optimization Strategy (MOST), Learn as You Go (LAGO), and Sequential Multiple Assignment Randomized Implementation Trial (SMART) designs that allow us to optimize implementation strategies and calibrate the level of implementation support provided based on demonstrated need [123–127].

Finally, we can avoid pseudoinnovation and efficiently develop the evidence base for strategies. One way is to improve reporting of implementation strategies, for more efficiently assessing the effectiveness of strategies with similar components and hypothesized change mechanisms. In some intervention science domains, integration of findings from different programs, teams, and studies is facilitated by the identification of intervention commonalities [128]. A similar approach could synthesize “common elements” of distinct implementation strategies, so they can be flexibly applied to a range of implementation challenges [129]. Another promising approach is the “meta-laboratory” described by Grimshaw and colleagues [6] that compares different ways of providing audit and feedback. The more effective approach quickly becomes the standard of care within the health system and is compared in a subsequent trial to another audit-and-feedback strategy that may offer efficiency or effectiveness improvements. This approach may be an efficient way of developing a robust evidence base for implementation strategies.

## Conclusion

We are privileged and humbled to be a part of a developing field and optimistic that it has a long and successful future. Aligned with the themes from our pre-mortem exercise, we confirm the importance of examining our assumptions, reflecting with humility, and planning the way forward as the field approaches 20 years since the launching of *Implementation Science*. Developmentally, we believe that the time is ripe to begin reflecting as a field. A key insight gleaned from our group is that implementation science drew us from other disciplines given its promise for enhancing population health and promoting health equity, but we find it is not immune from the threats that challenge other fields including intervention science, such as academic incentive structures and misalignment with collaborator timelines. The themes offered largely align with previous self-critical assessments from international teams outside of the US and also offers new perspectives. Synthesizing threats and opportunities from the perspective of international teams collectively is an important future activity and could be a focus of an international convening of implementation scientists [4, 5].

We see several key opportunities to enhance the future of the field and leverage the power of prospective hindsight to ensure our success. We wrote this piece as a conversation starter. We hope it generates reflection from the vantage point of other implementation partners, particularly implementation practitioners and international colleagues as our field continues to develop.

## Supplementary Information

**Additional file 1.** Details about the pre-mortem exercise.

## Data Availability

Not applicable.

## Abbreviations

NIH: National Institutes of Health
EBP: Evidence-based practice
SIRC: Society for Implementation Research Collaboration
ERIC: Expert Recommendations for Implementing Change
MOST: Multiphase Optimization Strategy
LAGO: Learn as You Go
SMART: Sequential Multiple Assignment Randomized Implementation Trial
