# Supplementary material for: Promises and pitfalls in implementation science from the perspective of US-based researchers: learning from a pre-mortem
Source: Implement Sci. 2022 Aug 13;17:55. doi: 10.1186/s13012-022-01226-3 (PMC9375077; doi:10.1186/s13012-022-01226-3)
Supplement: Supplementary file 1 — Additional file 1. Details about the pre-mortem exercise. [file 13012_2022_1226_MOESM1_ESM.docx]

**Additional file 1. Details about the pre-mortem exercise.**

We completed a “pre-mortem” virtually over 3 hours in January 2021 to reflect on the field’s advancements thus far and pitfalls or threats that could stymie progress. A pre-mortem uses prospective hindsight—a group imagines a failure and generates an explanation for it—to reduce the likelihood of the failure. To begin, we shared this prompt with our group: “NIH has prioritized and invested in implementation science to increase the impact of discovery and to reduce the research to practice gap. The year is 2025. The NIH Director has convened a meeting of Institute Directors and stated that the millions of dollars invested in implementation science studies in the past decade have not moved the needle and they want to deprioritize funding. What went wrong and why?”

We discussed the prompt in two groups of five and as a larger group to generate consensus on key themes about potential threats and opportunities. While structured on the prompt, the exercise was organically driven and we did not employ strict qualitative methods for empirical analysis. Following the exercise, RSB and MBLF, who respectively led the two small groups and larger group discussion, reviewed all notes and summaries and derived key themes and summaries. This information was shared with all participants to ensure that the interpretation appropriately reflected the discussion and conclusions (i.e., member-check). We approached this exercise with respect and humility, encouraged by the field's promise and motivated to ensure its success.
